# A Comparative Evaluation of a Novel Vaccine in APP/PS1 Mouse Models of Alzheimer's Disease

**DOI:** 10.1155/2015/807146

**Published:** 2015-02-11

**Authors:** Iván Carrera, Ignacio Etcheverría, Lucía Fernández-Novoa, Valter Ruggero Maria Lombardi, Madepalli Krishnappa Lakshmana, Ramón Cacabelos, Carmen Vigo

**Affiliations:** ^1^Department of Neuroscience, EuroEspes Biotechnology, Poligono de Bergondo, Nave F, 15165 A Coruña, Spain; ^2^Department of Neurobiology, Torrey Pines Institute for Molecular Studies, 11350 SW Village Parkway, Port Saint Lucie, FL 34987, USA; ^3^EuroEspes Biomedical Research Center, Institute for CNS Disorders and Chair of Genomic Medicine, University of Camilo Jose Cela, 15165 Madrid, Spain; ^4^Atlas Pharmaceuticals, Sunnyvale, CA 94089, USA

## Abstract

Immunization against amyloid-beta-peptide (A*β*) has been widely investigated as a potential immunotherapeutic approach for Alzheimer's disease (AD). With the aim of developing an active immunogenic vaccine without need of coadjuvant modification for human trials and therefore avoiding such side effects, we designed the A*β*_1–42_ vaccine (EB101), delivered in a liposomal matrix, that based on our previous studies significantly prevents and reverses the AD neuropathology, clearing A*β* plaques while markedly reducing neuronal degeneration, behavioral deficits, and minimizing neuroinflammation in APP/PS1 transgenic mice. Here, the efficacy of our immunogenic vaccine EB101 was compared with the original immunization vaccine cocktail A*β*_42_ + CFA/IFA (Freund's adjuvant), in order to characterize the effect of sphingosine-1-phosphate (S1P) in the immunotherapeutic response. Quantitative analysis of amyloid burden showed a notable decrease in the neuroinflammation reaction against A*β* plaques when S1P was compared with other treatments, suggesting that S1P plays a key role as a neuroprotective agent. Moreover, EB101 immunized mice presented a protective immunogenic reaction resulting in the increase of A*β*-specific antibody response and decrease of reactive glia in the affected brain areas, leading to a Th2 immunological reaction.

## 1. Introduction

Alzheimer's disease (AD) is the major cause of dementia worldwide and the development of effective therapies remains a major unmet medical need. It is known that it is a heterogeneous and complex disorder in which hundreds of genes distributed across the human genome might be involved in close cooperation with environmental factors and epigenetic phenomena leading to cerebrovascular dysfunction [1]. The resulting cognitive impairment is mainly associated with neuronal degeneration at the hippocampal dentate gyrus and entorhinal cortex, among other brain regions implicated in cognitive function, involved in learning and memory processes [2]. Based on amyloid cascade hypothesis, elevated levels of A*β* have been correlated with cognitive decline, mediating initial pathogenic events in AD dementia [3, 4] such as the accumulation of senile plaques and plaque-associated dystrophic neurites in the brain [5, 6]. Consequently, a progressive neuroinflammatory reaction has been observed surrounding A*β* plaques, resulting in astrogliosis and microglial activation. This inflammatory process may independently lead to neural dysfunction and cell death, thus establishing a self-perpetuating vicious cycle, which further contributes to neurodegeneration and enhances the pathological hallmarks of the disease [7]. Assuming that the neuropathological pathway defined by the amyloid cascade hypothesis causes AD, suppression of A*β* in the brains of patients in the early phase of dementia should become a primary therapeutic target. In this case, A*β* immunotherapies would become the most promising preclinical strategies as they have been proven to enhance clearance of A*β* in the brain of mice models.

In order to investigate new therapeutic strategies in AD, APPswe/PS1ΔE9 (APP/PS1) mice which overexpress the Swedish mutation of APP together with PS1 deleted in exon 9 that rapidly accumulates A*β* plaques at 6 months of age [8, 9] have been extensively used in AD research [10]. APP/PS1 mice also develop behavioral and learning deficits [11], plaque-associated neuritic abnormalities [12], inflammation reflected in activated microglia and astrocytes surrounding the A*β* plaques [13], and deficits in the pre- and postsynaptic cholinergic transmission [14]. In the last decade, these transgenic mouse models were extensively used in preclinical studies of active immunization [15, 16] with preaggregated A*β*, showing high anti-A*β* antibody titers in plasma, dramatic reduction of cerebral A*β* burden, and reduction in cognitive decline [17, 18]. These results in mice did not translate well in humans. Clinical trials conducted by Elan/Wyeth in 2001, using A*β* peptide delivered in QS-21 adjuvant (AN1792), resulted in a meningoencephalitis reaction in 6% of the treated patients. The trial then was immediately stopped. Subsequent studies suggested that these adverse events had been initiated by activation of cytotoxic T cells and/or autoimmune reactions [19–23]. Recently, we reported that immunotherapeutic treatment with EB101 vaccine, consisting of A*β*_1–42_ delivered in a novel immunogen-adjuvant, composed of liposomes-containing phosphatidylcholine, phosphatidylglycerol, cholesterol, and sphingosine-1-phosphate (S1P), results in a marked reduction of A*β* plaque burden and dystrophic plaque neurite density, diminution of astrocytosis, and attenuation of amyloidosis-induced inflammation [24, 25]. In the present study we compare the efficacy of EB101 versus the original immunization vaccine cocktail (A*β*_42_ + CFA/IFA) [17] in the APP/PS1 mouse model before and after the onset of A*β* pathology. Moreover, we also characterized the effect of S1P in the immunotherapeutic response of EB101 in this mouse model, showing that it plays a key role as a regenerative agent in the central nervous system [26]. The findings presented were obtained by using immunocytochemistry techniques, neuronal anatomic mapping, and sera antibody/cytokines detection by ELISA and motor behavioral tests, suggesting a notable effectiveness of EB101 over A*β*_42_ + CFA/IFA vaccine in clearing A*β* plaques, reducing dystrophic plaque neurites, preventing inflammation in the entorhinal cortex and hippocampus in this transgenic mouse model. These results warrant further studies, which could prove that EB101 is a promising vaccine to treat AD patients avoiding adverse effects.

## 2. Materials and Methods

### 2.1. Animals

A well-studied mouse model of A*β* amyloidosis is the double-transgenic mice B6C3F1/J (APPswe/PS1dE9), expressing a chimeric mouse/human amyloid precursor protein (Mo/HuAPP695swe) and human presenilin 1 (PS1-ΔE9) mutants, both directed to central nervous system (CNS) neurons, that exhibits A*β* plaques in the hippocampus and cortex beginning at 6 months of age (Jackson Laboratory, Bar Harbor, ME). All experimental procedures were conformed to the guidelines established by the European Communities Council Directive (86/609/EEC), the EU Directive 2010/63/EU, and the Spanish Royal Decree 1201/2005 for animal experimentation and were approved by the Ethical Committee of the EuroEspes Biotechnology Research Centre (Permit number: EE/2012-344).

### 2.2. Experimental Design

**Figure 1:**
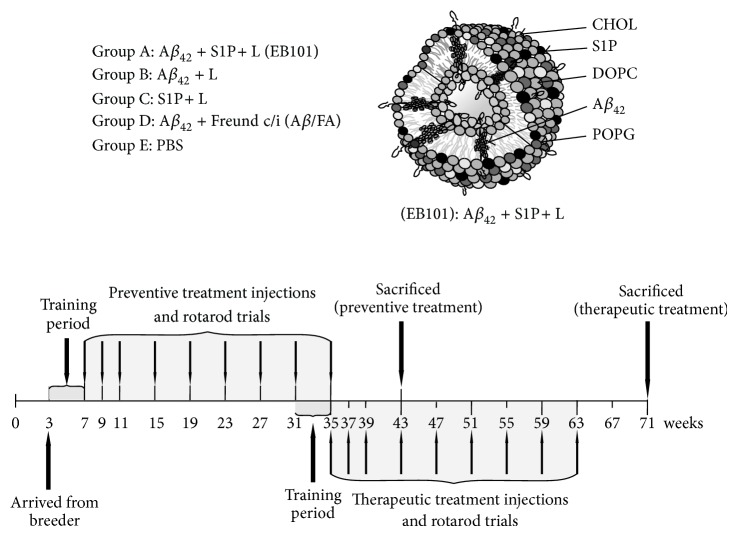
Biophysical characterization of the EB101 vaccine and experimental design. Biophysical structure of A*β*_42_/S1P-containing liposome (EB101) and schedule of experiments.

Two groups of experimental studies, preventive treatment (before amyloid deposition onset, starting at 7 weeks of age) and therapeutic treatment (after amyloid deposition onset at 35 weeks of age), were carried out as described in recent reports [24, 25] similar to the long-term protocol reported by Schenk and colleagues [17] and represented in Figure 1. Mice of both sexes (balanced between treatment groups) were randomly assigned to each of the two experimental groups, and they were divided into five treatment groups per study. Preventive treatment: Group A was formed by 20 mice (14 transgenic and 6 wild-type mice; 14 + 6) that were immunized with a cocktail of synthetic human A*β*_42_/S1P-containing liposomes (EB101); Group B was formed by 20 mice (14 + 6), immunized with A*β*_42_/liposome without S1P; Group C was formed by 20 mice (14 + 6) immunized with S1P-containing liposomes; Group D was formed by 20 mice (14 + 6) immunized with A*β*_42_/Freund's adjuvant complete-incomplete (A*β*_42_ + CFA/IFA); and Group E was formed by 16 mice (12 transgenic and 4 wild-type mice, control) inoculated with PBS. Therapeutic treatment: the same treatments were administrated in Groups A, B, C, and D, formed by 20 mice in each group, while the Group E was formed by 16 mice inoculated with PBS, as control. Mice were immunized with nine injections for seven months, inoculating 100 *μ*L per injection containing a cocktail of A*β* (100 *μ*g) and phospholipid mix (1 mg) (Figure 1).

### 2.3. Experimental Procedure

The methodological procedures to prepare the EB101 and A*β*_42_ + CFA/IFA immunizations were strictly followed as described by Carrera et al. [24] and Webster et al. [10], respectively. In summary, the EB101 vaccine was prepared using 2 mg of human A*β*_1–42_ (TOCRIS bioscience; Tocris Cookson Ltd.), corresponding to the human form of the predominant amyloid A*β* found in the brains of patients with AD, dissolved in 0.9 mL water up to 1 mL by adding 0.1 mL of 10xPBS, then vortexed, lyophilized, and stored. Liposomes were prepared from 1,2-dioleoyl-sn-glycero-3-phosphocholine (DOPC), 1-palmitoyl-2-oleoyl-sn-glycero-3-phosphatidylglycerol, sodium salt (POPG), cholesterol (CH; Northern lipids INC.), and plus/minus D-erythro-sphingosine-1-phosphate (S1P) (AVANTI at 0.3/0.3/0.39/0.01, molar ratio, resp.) as described by Lang and colleagues [30]. To prepare the A*β*_42_ + CFA/IFA vaccine, A*β*_1–42_ was emulsified 1 : 1 (v/v) with complete Freund's adjuvant (CFA) for the first immunization, followed by a boost of incomplete Freund's adjuvant (IFA) monthly thereafter. Once the immunization period finished, mice were kept in the cage for 2 months before being euthanized for analysis. Mice were 11 months old at the end of preventive treatment and 18 months old at the end of therapeutic treatment.

### 2.4. Spleen Cell Preparation, Cytokine ELISAs, and Flow Cytometric Analysis

Mice splenocytes were harvested from a clamped spleen tissue, as described by Carrera et al. [24]. The B-cell-enriched suspensions were obtained from spleen cells that were depleted by selective adherence to glass Petri dishes for 2 h at 37°C. This procedure yielded an enriched B-cell population >90% CD19+ cells with <1% CD3+ cells and <5% CD11c+ cells, as determined by flow cytometry analysis, and >95% of viable cells, as determined by trypan blue exclusion. Cells were seeded into 96-well tissue culture plates at a density of approximately 5000 cells per well. After stimulation, 10 *μ*L of MTT solution was added to each well. Absorbance was measured on an ELISA plate reader with a test wavelength of 570 nm. The results obtained from triplicate assays were expressed as stimulation indexes (ratio between mean OD from stimulated and unstimulated cultures).

### 2.5. Measurement of Antibody Titers

Serum antibody titers were measured using methods described previously [24, 31]. Briefly, 96-well microtiter plates were coated with 1 *μ*g/mL of A*β*_1–42_ in well-coating buffer and incubated overnight at room temperature (RT). The sera were added to the wells at a starting dilution of 1/100 in specimen diluent (0.014 M sodium phosphate, pH 7.4, 0.15 M NaCl, 0.6% bovine serum albumin, 0.05% thimerosal). Four serial dilutions (1/400, 1/1,600, 1/6,400, and 1/25,600) of the samples were made directly in the plates in twofold steps to reach a final dilution of 1/102,400. After incubation, the second antibody (goat anti-mouse IgG conjugated to horseradish peroxidase from Boehringer Mannheim) was added to the wells (100 *μ*L/well at a dilution of 1/4,000 in specimen diluent) and incubated for one hour at RT. The chromogen reaction (3,3,5,5′-tetramethylbenzidine obtained from Pierce Chemicals) was stopped by the addition of 50 *μ*L 0.5 M H_2_SO_4_. The color intensity was then read on an ELISA plate reader (Bio-Rad 680 ELISA Reader). Titers were defined as the reciprocal of the dilution of serum giving one half the maximum OD. Maximal OD was generally taken from an initial 1/100 dilution.

### 2.6. Immunohistochemistry

Immunohistochemical data was analyzed by using methods described previously [24]. In summary, parallel sections were pretreated with H_2_O_2_ in phosphate-buffered saline at 37°C for 15 minutes to eliminate endogenous peroxidase, rinsed twice in 0.05 M Trizma buffered saline (TBS) containing 0.1% Tween-20 at pH 7.4 (TBS-T) for 10 minutes each, pretreated with blocking avidin/biotin kit and then incubated overnight with the primary antibodies as described in Table 1. The sections were successively rinsed in TBS-T, incubated in goat IgG anti-rabbit (Dako) or goat IgG anti-mouse (Dako), depending on the primary antibody, for 1 hour, rinsed in TBS-T, and then incubated for 30 minutes in ABC kit system (Vectastain; Vector). The labeling was revealed by incubating sections with 3,3-diaminobenzidine as chromogen and hydrogen peroxide as oxidant. In several adjacent sections, negative controls performed by omitting the primary, secondary, or tertiary antibodies showed no immunostaining. Prussian blue iron staining was performed to detect hemosiderin to reveal signs of microhemorrhage although no considerable perivascular microhemorrhages were observed in the brain sections analyzed.

### 2.7. A*β* Load Calculation

Digital images of 7 randomly selected microscopic transverse sections per treated animal were analyzed and the percentage of immunostained A*β* deposition was determined for all the markers studied, as defined by the stereotaxic Bregma coordinates. A total of 7 selected sections per animal were evaluated using the NIH Image J program by defining region of interest and setting a threshold to discriminate nonspecific staining. Quantitative analysis of amyloid burden area was also performed in the hippocampal regions and parietal/temporal cortical regions. We used area/pixels analysis software (Pixcavator 4), to quantify the average number of pixels inside each A*β* plaque per brain section. Therefore, Pixcavator intensity threshold imaging was set to mark only those areas stained with *β*-amyloid antibody (A*β* load) relative to the entire image area. The immunostaining intensity of each image was first averaged per mouse and then per group and expressed in percentage units.

### 2.8. Imaging

Images were visualized using a microscope (Olympus BX50) and digitized using a digital camera (DP-10; Olympus). A*β* burden data was determined by quantitative image analysis (Pixcavator 4) and reported as plaques per section (p/s) or percentage of immunolabeled area captured within total sample area. The microphotographs were adjusted for brightness and contrast with Corel Photo-Paint (Corel, Ottawa, Canada) and plates were composed with Corel Draw.

### 2.9. Sensorimotor and Cognitive Testing

After the administration of the vaccine, mice were subjected to sensorimotor and memory testing to measure their coordination and learning performances during the experimental period and to verify that any treatment-related effects observed in the memory tasks could not be explained by differences in sensorimotor abilities. Prior to testing, as previously described [32], the mice were adapted to the room and testing conditions.

#### 2.9.1. Rotarod

The motor strength, ability, balance, and coordination skills of all the experimental mice in the two treatment periods were evaluated in a rotarod apparatus (Columbus Instruments, Columbus, OH) operated at 10 rpm, beginning at 7 weeks of age (preventive group) and 35 weeks of age (therapeutic group). The animals were adapted to the apparatus by receiving training sessions during four weeks, sufficient to reach a baseline level of performance. This procedure was designed to assess motor behavior during six trials of a group/day. If the mouse remained on the rod for 1 minute, the test was completed and scored as 1. Each animal was tested previous to the treatment injection for three sessions, with each session separated by 15 minutes. The time that each mouse remained on the rod was registered automatically and stopped when the animal fell or inverted (by climbing) from the top of the rotating barrel. In this experiment, one investigator evaluated the rotarod performance and was blinded to mice treatments, and the second one treated the mice and scored the test to avoid a bias.

#### 2.9.2. Fear Conditioning/Active Avoidance

This fear-motivated associative avoidance test is based on electric current where the mouse has to learn to predict the occurrence of this aversive event by actively moving to a different compartment (escape platform). This active avoidance task provides a simple way to assess associative learning and memory by testing the ability of the mouse to avoid an aversive event in response to a stimulus cue. The measures recorded number of nonresponses (the mouse failing to reach the escape platform during the trial) and response latency (latency to avoid or escape) serves as an index of learning and allows memory to be assessed. After habituation for 5 min, mice received 30 randomized escapable footshocks at an intensity of 0.25 mA. In each shock trial, the time to reach the escape platform, located constantly at a central quadrant, from the start location was recorded as latency. Each trial lasted a maximum of 30 s. If the mouse failed to find the platform within 30 s, it was manually guided to the platform and allowed to stay for 10 s, the latency was recorded 30 s. Experimental results are indicated as mean response time for each treatment period.

### 2.10. Onset and Survival

Disease onset and progression were confirmed and defined by periodic mice sacrifice and neuropathological analysis. The mortality rates of APP/PS1 transgenic mice in the course of both sets of experiments were 42% for A*β*_42_ + CFA/IFA, 17% for EB101, and 12% for the vehicle.

### 2.11. Statistical Analyses

For statistical analyses, SPSS (version 11.0; SPSS Inc, Chicago) was used and a *P* value < 0.05 was considered statistically significant. The correlation average of A*β* plaque density, burden area, and antibody titers were analyzed and compared in multiple groups by one-way analysis of variance (ANOVA), followed by Fisher's LSD* post hoc* means comparison test analysis when relevant. All graphs display group mean ± SEM.

### 2.12. Limitations of the Study

The ultimate goal of the AD preclinical research is developing a therapeutic drug strategy in the ideal animal model that should recapitulate most if not all the neurobehavioral pathology and underlying the same mechanisms described in human AD, such as the rate of amyloid plaque accumulation, neurodegeneration, and behavioral and cognitive alterations. Although such an ideal mouse model has not yet been generated, we believe that APPswe/PS1ΔE9 transgenic mice present enough pathological features to answer the specific questions addressed by the present study. Active A*β* immunotherapy, in which antibodies are produced by the individual upon contact with the antigen, has great potential for preventive AD therapy as shown here in animal models, although some aspects such as an undesirable immune response induced by the adjuvant, long immune response adjustment, age-dependent effects, inflammation, and microhemorrhages should be addressed before its application in clinical trials. Being aware of these issues, we designed the present study aiming to solve and overcome all these disadvantages.

## 3. Results

### 3.1. Efficacy of EB101 versus A*β*_42_ + CFA/IFA in Preventing A*β* Burden Development

**Figure 2:**
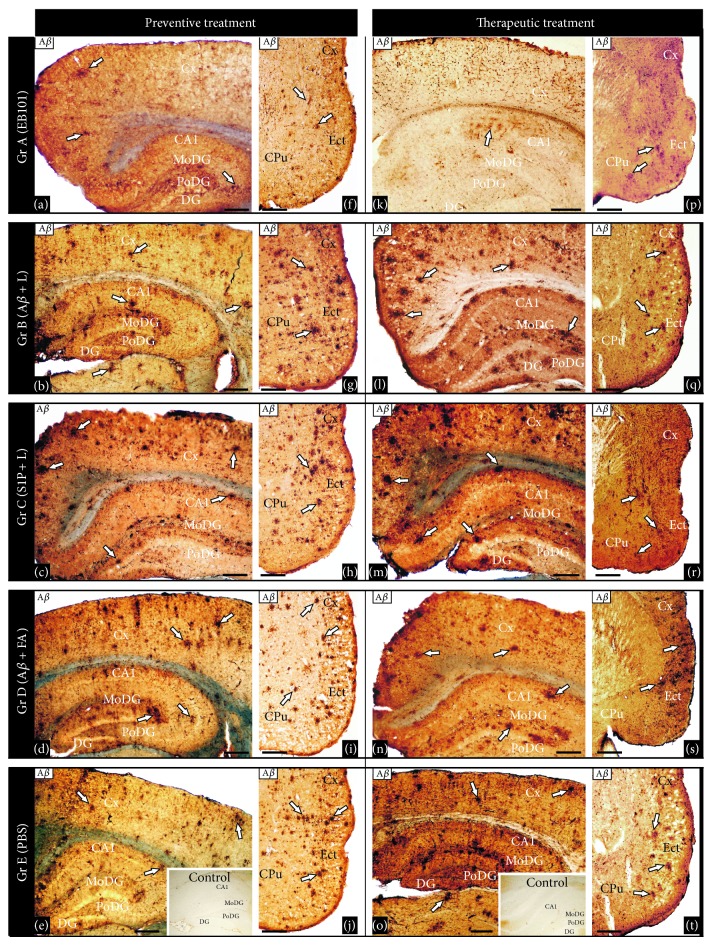
Comparative effect of EB101 vaccine on beta amyloid deposits. Comparative photomicrographs of A*β* immunoreactivity were taken in the hippocampus ((a)–(e), (k)–(o)) and cortical ((f)–(j), (p)–(t)) brain regions of transgenic mice with different treatments before A*β* plaques development, preventive treatment ((a)–(j)) and after the A*β* plaques developed, therapeutic treatment ((k)–(t)). Preventive treatment: transverse brain sections of 11-month-old mice show reduced number of A*β* deposits in the dentate gyrus, hippocampal subregion CA1 (a), and entorhinal cortex (f) following EB101 vaccine immunization (Group A), contrasting with the numerous A*β* immunoreactive plaques in the correspondent mouse brain sections of Groups B ((b), (g)), C ((c), (h)), D ((d), (i)), and E ((e), (j)). Immunoreactive A*β* in the brain sections of wild-type (WT) mice is absent (inserts in panels (e), (o)). Therapeutic treatment: transverse brain sections of 18-month-old mice are shown in panels (k)–(t). Treatment with EB101 almost completely cleared A*β* load in the dentate gyrus and reduced notably the density in hippocampal subregion CA1 (k) and entorhinal cortex (p) compared to the same areas of mouse Groups B ((l), (q)), C ((m),(r)), D ((n), (s)), and E ((o), (t)), as determined by number of plaques and staining intensity area. Scale bar: 100 *μ*m.

**Figure 3:**
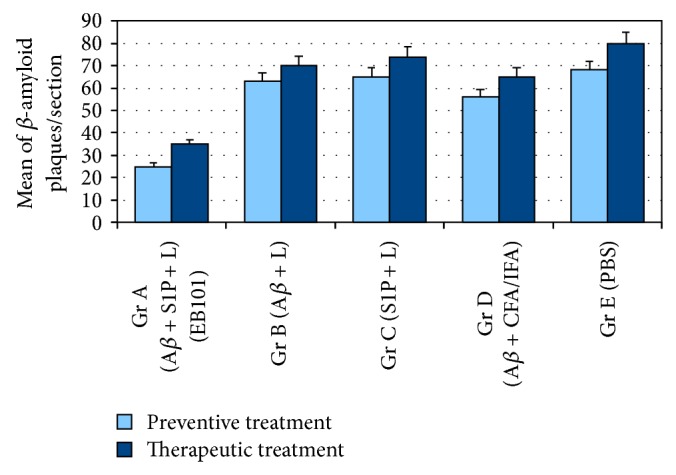
Comparative analysis of A*β* plaque burden in the brains of APP/PS1 mice. Mean of A*β* plaque burden in the hippocampal and cortical regions of APPswe/PS1ΔE9 mice in the five treatment groups. The mean of the A*β* burden is significantly reduced (*P* < 0.05) in Group A (EB101) when compared with Groups B–E during preventive treatment and markedly reduced in the therapeutic treatment period. Data are presented as mean ± standard error of the mean (SEM). Density mean of A*β* plaques in Tg mice hippocampus.

**Figure 4:**
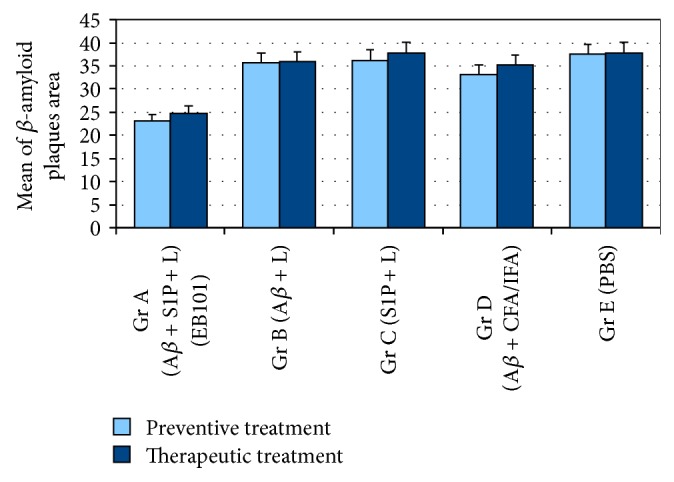
Comparative quantification of A*β* burden area in the brain of APP/PS1 mice. Quantitative analysis of A*β* burden area in the hippocampal and cortical regions of APPswe/PS1ΔE9 mice treated with EB101, A*β*/liposomes, S1P/liposomes, A*β*_42_ + FA/IFA, and PBS, represented by the number of pixels inside the stained area of each A*β* plaque. This graphic shows that A*β* plaques of EB101-treated mice (Group A) are significantly smaller in size (*P* < 0.05) than those of the other four treatment groups. Data are presented as mean ± SEM. Mean of A*β* plaques area in Tg mice hippocampus.

Recently, we demonstrated that immunization with EB101 vaccine prevents and reverses the AD neuropathology by the marked reduction of A*β* plaques, plaque-associated dystrophic neurites, and astrocytosis in mice brain. Now, we conducted comparative immunization studies between EB101 and A*β*_42_ + FA/IFA vaccines in the prevention of A*β* deposits in the mouse brain. A*β* plaques and vascular amyloid loads were detected, counted, and measured as the percent of total surface stained by monoclonal anti-A*β* antibody in the hippocampal and cortical sections. The plaque amyloid load observed in the representative photomicrographs presented in Figure 2 shows a significant difference across the five experimental groups (ANOVA, *F* = 3.15, *P* < 0.001). As a result of the first experimental phase, based on preventive treatment, we observed that hippocampal load was significantly reduced in the EB101 immunized mice (A, Figures 2(a) and 3) when compared with different vaccine components (B-C, Figures 2(b)-2(c) and 3) and moderate compared with A*β*_42_ + CFA/IFA vaccine (D, Figures 2(d) and 3). In fact, the load in these treated groups (B-C) was not significantly different from PBS treated mice (E, Figures 2(e) and 3), although there is a positive correlation between the presence of A*β* in treatment regime and the total A*β* burden (Figures 2(b), 2(d), and 3). In the EB101 treated mice, a few compacted A*β* plaques were observed in retrosplenial granular region of the cortex and at the polymorph dentate gyrus of the hippocampus (Figure 2(a)). Strikingly different was the distribution of A*β* plaques observed in the other treatment groups where numerous A*β* plaques were located at different cortical and hippocampal layers (Figures 2(b)–2(e)). This data was also supported by total A*β* plaques density and size quantification made by image analysis software in hippocampal sections stained with anti-A*β* antibody (Figures 3 and 4). The data obtained in the present study validate our previous observations, demonstrating that the mean burden A*β* plaques (Figures 2(a) and 3) of Group A (25 ± 5 p/s) were significantly different from the other treated groups (63 ± 6 p/s in Figure 2(b); 65 ± 6 p/s in Figure 2(c); 56 ± 5 p/s in Figure 2(d); 68 ± 6 p/s in Figures 2(e) and 3). This represents a decrease in the mean percentage of A*β* plaque area of 30.1–38.2% in the EB101 immunized mice group relative to other tested groups (Figures 2(a)–2(j), and 4). The appearance of amyloid plaques in the entorhinal (Figures 2(f)–2(j)) and piriform cortex was also analyzed, being detected in low density in Group A (Figure 2(f)), moderate density in Groups B (Figure 2(g)) and D (Figure 2(i)), and high density in the entorhinal cortex of Groups C (Figure 2(h)) and E (Figure 2(j)). Therefore, this data shows that, in the preventive treatment period, the clearance effect of the EB101 vaccine on A*β* plaques per section was about 64% (Figures 2(a) and 2(f)) compared to positive controls, while in the other treatments regimes 19% was detected in A*β*_42_ + CFA/IFA (D), 10% in A*β*_42_/liposome without S1P (B), and 7% in liposome with S1P treated mice (C). Photomicrographs show that, in all groups, these plaques were mainly localized in the external layers of the entorhinal cortex (Figures 2(f)–2(j)). In addition, we have observed lower stained intensity of the A*β* plaques in the brain regions of EB101 treated mice, which contrasts markedly with the intensely stained A*β*-immunoreactivity of these plaques in each brain section of the other experimental mice groups.

### 3.2. Efficacy of EB101 versus A*β*_42_ + CFA/IFA in Clearing A*β* Plaque Burden

In a second set of experiments, we compared the therapeutic effect of EB101 vaccine with A*β*_42_ + CFA/IFA in the reduction of A*β* in the brain after the plaques were established in the 35-week-old APP/PS1 transgenic mice (Figures 2(k)–2(t)). The mice were treated and analyzed using the same protocol as in the preventive treatment. To determine whether EB101 vaccine attenuates and/or reverses the massive development of *β*-amyloid plaques, brain sections from wild-type and transgenic mice of all experimental groups were immunostained with the specific antibody recognizing A*β*_1–42_ epitopes. These results show that A*β* deposits (Figures 2(k)–2(t)) were notably reduced in brain sections of the EB101 treated mice (A), markedly different from the A*β* burden levels observed in the other treated groups (B–E). As observed in the corresponding photomicrographs, EB101 treated mice showed a few compacted A*β* plaques mainly located at the CA1 layer of the hippocampus (Figure 2(k)). No reduction in plaque density was observed in the other experimental groups (Figures 2(l)–2(o)), where numerous large compacted A*β* plaques were located in almost every layer of each brain section. The most densely populated section with A*β* plaques were those with no A*β*_1–42_ treatment as shown in Groups C (Figure 2(m)) and E (Figure 2(o)), while a slight reduction of this hallmarks was observed in mice Groups B (Figure 2(l)) and D (Figure 2(n)). The hippocampal sections of the therapeutic treatment with EB101 vaccine showed a similar pattern as that observed in the preventive treatment. The mean burden of A*β* plaques in Group A (35 ± 4; Figure 3) was significantly different from the other treated groups (70 ± 5 in B; 74 ± 4 in C; 65 ± 5 in D; 80 ± 6 in E). The A*β* plaque area in the EB101 immunized mice group was reduced by 29.7–34.5%, relative to the other tested groups (Figure 4). As observed in the preventive set of experiments, this data show that in the therapeutic treatment period, the clearance effect of the EB101 vaccine on A*β* plaques per section was about 59% compared to controls. While in mice group treated with A*β*_42_ + CFA/IFA the clearance effect was about 20% in Group D, 13% in Group B, and 8% in Group C. The same pattern of A*β*-immunoreactivity was observed in the entorhinal cortex when comparing all experimental treatment groups (Figures 2(p)–2(t)). Only a few A*β* plaques were observed in the external layer of the entorhinal and piriform cortex of mice treated with EB101 (Figure 2(p)), while numerous plaques were observed in the corresponding section of Groups B (Figure 2(q)) and D (Figure 2(s)), which show a moderate density of A*β* plaques, while Groups C (Figure 2(r)) and E (Figure 2(t)) show densely distributed plaques along the medial and external layers of the entorhinal-piriform cortex. As described in the preventive treatment, we also detected a reduced staining intensity of the A*β* plaques in the brain regions of mouse Group A, contrasting with the intense A*β*-immunoreactivity of plaques in the other experimental mouse groups as shown in the photomicrographs of Figures 2(k)–2(t).

### 3.3. Immune Response Effects of EB101 versus A*β*_42_ + CFA/IFA Vaccine

**Figure 5:**
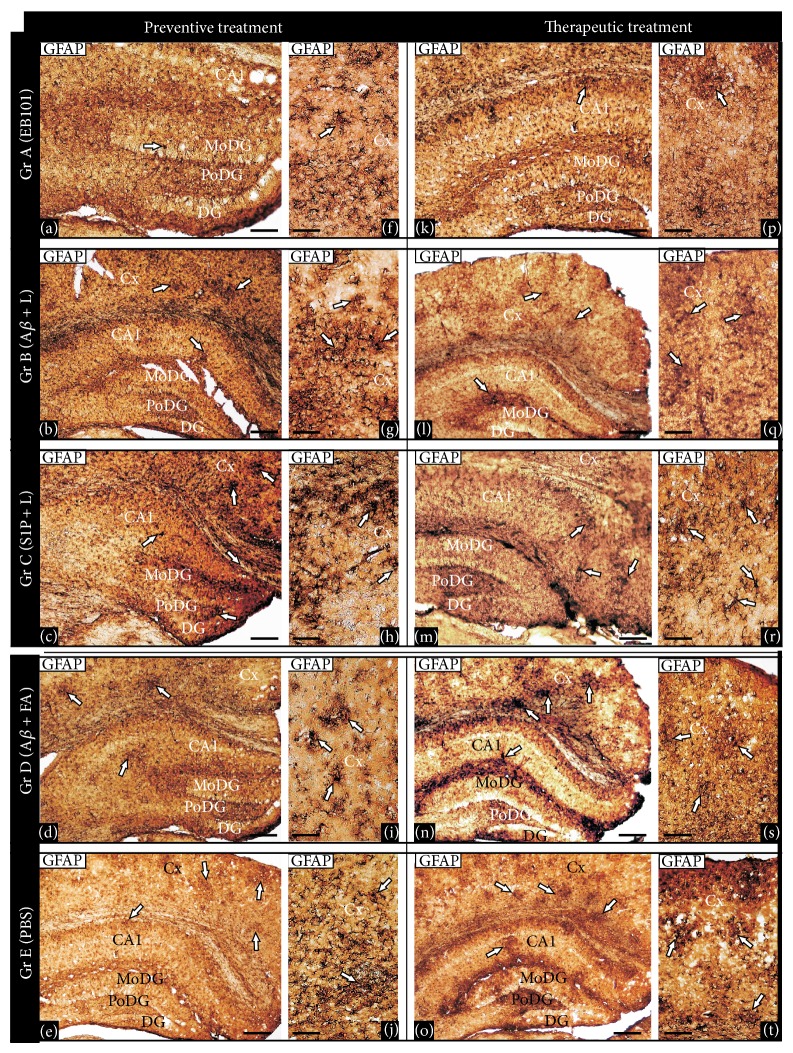
Comparative effect of EB101 vaccine on astrocytosis. Comparative photomicrographs of GFAP immunoreactivity in the hippocampus ((a)–(e), (k)–(o)) and cortical ((f)–(j), (p)–(t)) brain regions at the preventive treatment phase ((a)–(j)) and therapeutic treatment group ((k)–(t)). Preventive treatment: transverse brain sections of 11-month-old mice treated with EB101 show an almost complete prevention of astrocytosis in the detailed sections of the dentate gyrus (a), contrasting with numerous dystrophic reactive astrocytes, typical of an inflammatory reaction, observed in different hippocampal corresponding areas of mouse brains in Group B (A*β*/Lip-treated mice; (b)), Group C (Lip/S1P-treated mice; (c)), Group D (A*β*_42_ + CFA/IFA-treated mice; (d)), and Group E (PBS-treated mice, (e)). Immunoreactive astrocytosis in the brain sections of WT mice is absent (data not shown). Detailed sections of the parietal cortex of treated mice show a gradient density of reactive astrocytes immunoreactive to GFAP, indicating a neuroinflammation pattern. Comparative cortical sections show numerous astrocytosis areas in mice Groups C and E ((h), (j)) and wide reactive astrocytes clusters in mice Groups B and D ((g), (i)), while just a few of them are observed in Group A (f). Therapeutic treatment: transverse brain sections of 18-month-old mice of Group A show a notable reduction of astrocytosis in the hippocampal region (k) after EB101 vaccine immunization. Note the astrocytosis contrast density between EB101-treated mice and the other treated groups ((l)–(o)), where numerous immunoreactive GFAP clusters are observed in the correspondent brain sections. A few immunoreactive GFAP clusters are observed in the parietal cortex of EB101 treated mice group, while a moderate ((q), (s)) to high ((r), (t)) density of these neuropathological inflammation clusters is apparent in the other treatment groups (Groups B–E). For abbreviations, see list. Scale bar: 100 *μ*m.

Astrocyte activation implicated in neuroinflammation, amyloidogenesis, and neuronal cell death in AD was compared after treatment of the transgenic mice with EB101 and A*β*_42_ + CFA/IFA. Immunohistochemical analysis of GFAP-reactive cells was carried out in the brains of the treated mice in the preventive and therapeutic studies (Figure 5). After the preventive treatment (Figures 5(a)–5(j)), EB101 significantly reduced the density of GFAP-reactive cell clusters in the hippocampal and cortical sections as compared with A*β*_42_ + CFA/IFA (D), A*β*/Lip without S1P (B), Lip with S1P (C), and PBS (E). The distribution of GFAP immunoreactive cells in the transverse section of the mouse brains treated with EB101 shows a few scattered GFAP-reactive clusters, mainly at the CA1 hippocampal layers, contrasting with numerous dystrophic reactive astrocytes observed in different hippocampal areas of mouse brains in Groups B–E (Figures 5(b)–5(e)). Similar neuroinflammation pattern was observed in the retrosplenial cortical layers, where a few GFAP-reactive clusters were observed in EB101 treated mice (Figure 5(f)) compared with their profuse presence in the other experimental groups (Figures 5(g)–5(j)). After therapeutic treatment, the GFAP immunoreactivity distribution pattern in mouse brains treated with EB101 was similar to control mice (wild-type), mostly devoid of reactive GFAP clusters, except a few scattered ones in the CA1 hippocampal external layers (Figure 5(k)). In contrast, there is a moderate density in Group D (Figure 5(n)) and extensive density in Group B (Figure 5(l)), Group C (Figure 5(m)), and Group E mice (Figure 5(o)). Photomicrographs of retrosplenial cortex show similar neuroinflammation pattern, where a few GFAP clusters detected in the EB101 treated mice (Figure 5(p)) contrast with a more extensive density of clusters in the other groups (Figures 5(q)–5(t)). No astrocytosis was observed in wild-type mice during preventive or therapeutic treatment.

### 3.4. Comparative A*β* Antibody Titers and Th1/Th2 Cytokine Levels in Treated Mice

**Figure 6:**
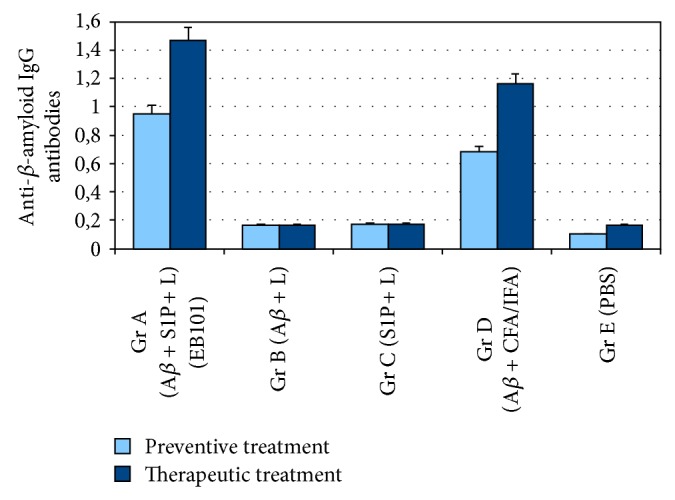
Quantification of specific anti-*β*-amyloid IgG in immunized APP/PS1 mice. The presence of anti-*β*-amyloid antibodies measured in the sera of vaccinated and control mice groups during the preventive and therapeutic treatments indicates that EB101 liposome vaccine was able to induce the highest production of specific IgG antibodies. Compared to the Group D immunized mice, the level of antibodies observed in the Group A was also higher, suggesting a more effective immune response, (*P* < 0.01 versus Group D; *P* < 0.001 versus Groups B, C, and E). Quantification of specific anti-*β*-amyloid IgG in immunized Tg mice.

**Figure 7:**
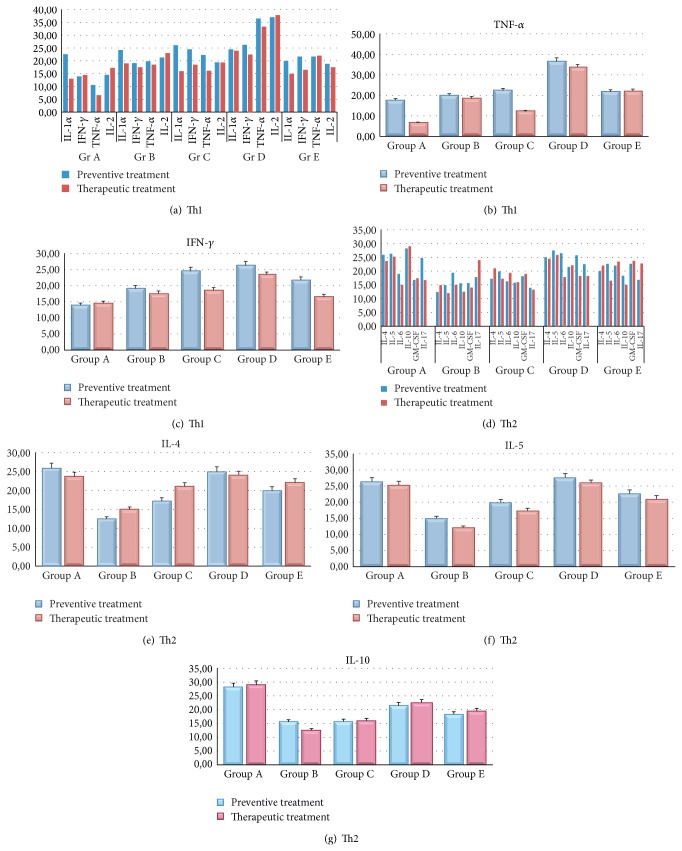
Detection of Th1 and Th2 cytokine profile. Measurements of the effect of EB101 and A*β*_42_ + CFA/IFA vaccines on Th1 and Th2 cytokines profile during preventive and therapeutic treatments. ((a)–(c)) A significant increase of Th1 cytokine profile was observed in preventive treatment in Group D with respect to all other groups, while a tendency to a reduction in Th1 secretion was observed in Group A with respect to all other groups in both preventive and therapeutic treatment. ((d)–(g)) The changes in Th2 protein levels were observed in the IL-4, IL-5, and IL-10 cytokine secretions of Group A (*P* < 0.05 versus Group D; *P* < 0.01 versus Group E).

A*β* antibody titers in the sera were determined by ELISA during mouse immunization (Figure 6). Preventive treatment with EB101 resulted in a marked increase of specific IgG A*β*_1–42_ antibody production in all animals. Mice in A and D groups developed and maintained serum antibody titers between 1 : 2000 and 1 : 8000. Mice in Groups B, C, and E showed no significant anti-A*β* antibody production. Antibody production and titer in Group B were three and four times lower than those of Groups D and A, respectively. After therapeutic treatment all mice treated with EB101 and A*β*_42_ + FA/IFA produced high levels of IgG antibodies. Titer levels of Groups A and D were significantly increased to those detected after preventive treatment (*P* < 0.01 versus Group D; *P* < 0.001 versus Groups B, C, and E), although antibody level in Group D was 1/3-fold lower than in Group A (Figure 6). The effect of EB101 (Group A) and A*β*_42_ + CFA/IFA (Group B) vaccines on Th1 and Th2 cytokines was also studied (Figure 7). Measurements of Th1 and Th2 cytokines in sera of immunized transgenic mice indicate that significant changes occurring in protein levels of Th1-related inflammatory mediators following preventive (Figures 7(a)–7(c)) and therapeutic (Figures 7(d)–7(g)) treatment with EB101 vaccine. A significant increase of Th1 cytokine profile was observed in preventive treatment in Group D, with respect to all other groups, mainly reported by the high levels of TNF-*α* (Figure 7(b)) and IFN-*γ* (Figure 7(c)) cytokine secretions, while due to the high mortality rate during the therapeutic treatment no significant data was obtained from this mice group. In addition, a tendency to Th1 secretion reduction was observed in Group A with respect to all other groups in both preventive and therapeutic treatment. The changes in Th2 protein levels (Figure 7(d)) followed the inverse trend, where the highest differences between Group A and the other treatment groups were observed in the IL-4, IL-5, and IL-10 cytokine secretions (*P* < 0.05 versus Group D; *P* < 0.01 versus Group E; Figures 7(e)–7(g)).

### 3.5. Comparative Motor Coordination and Memory Performances

**Figure 8:**
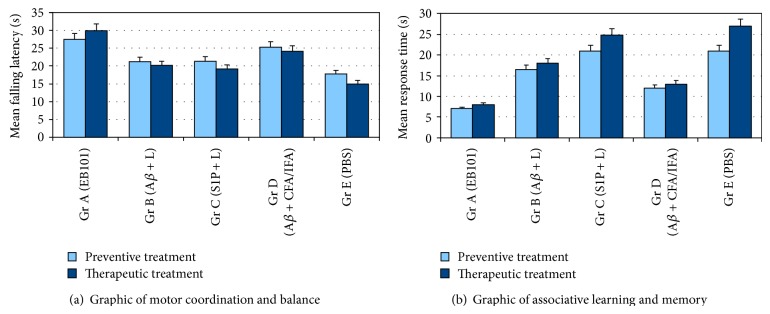
Analysis of motor coordination and balance. (a) Rotarod results of motor coordination and balance test after the preventive and therapeutic treatment showing improved effect of EB101 and A*β*_42_ + CFA/IFA-vaccine compared to other treated mice groups (*P* < 0.05) and (*P* < 0.02) versus control mice. Latency to fall on the rotarod apparatus was significantly improved in EB101 immunized mice. Values represent means of all six trials of a group/day. (b) Spatial learning and memory tested by fear conditioning-active avoidance test. The mean response time of each treatment group to reach the platform is represented. EB101 and A*β*_42_ + CFA/IFA immunized mice had significantly lower latencies than the other treatment groups (*P* < 0.01) versus control mice.

In order to validate the locomotion integrity during the entire immunotherapeutic process as reported in similar previous studies, motor coordination tests have showed that EB101-treated mice spent significantly more time on the accelerating rotarod. This motor coordination task shows that EB101 immunization affected positively their performance (Figure 8(a)). A*β*_42_/liposome (B), S1P-containing liposomes (C), and PBS (E) (*P* < 0.006) mice performed poorly in comparison to EB101 mice (*P* < 0.05) or A*β*_42_ + CFA/IFA mice (*P* < 0.02) in motor abilities and coordination. The fear conditioning-active avoidance test was used to assess the spatial learning and memory functions. As expected, both EB101 and A*β*_42_ + CFA/IFA immunized mice readily learned the location of the platform, as shown by a decrease in the escape latency over training days, while the control group mice exhibited no significant changes during 6-day trials. The average escape latencies of EB101 (6.8 ± 2 s, 7.61 ± 2.2 s) and A*β*_42_ + CFA/IFA (11.6 ± 3.3 s, 12.3 ± 2.1 s) immunized mice at the end of preventive and therapeutic periods, respectively, during the consecutive 6 days trial, were significantly shorter than the other treatment groups (A*β*_42_/liposome: 16.3 ± 3.1 s, 17.5 ± 2.8 s; S1P-containing liposomes: 20.2 ± 1.9 s, 24.8 ± 2.7 s) and the control (21.16 ± 3.8 s, 26.8 ± 2.2 s) (*P* < 0.01). There was no significant difference between EB101 and A*β*_42_ + CFA/IFA immunized group (*P* > 0.05) (Figure 8(b)). These results showed that spatial learning was improved in these immunized mice.

## 4. Discussion

In this study, we have compared a novel active A*β* immunotherapy, the EB101 vaccine, against A*β*, with the A*β*_42_ + CFA/IFA vaccine, that was first taken into clinical trials with coadjuvant Qs-21 modification (AN1792 by Orgogozo et al. [19]). This type of vaccine design was thought to induce a strong T-cell mediated inflammatory response (IL-2 and IFN-c positive responses indicative of a Class II (CD4) Th-1 type response [33], leading to acute meningoencephalitis in the 6% of the patients). The EB101 active immunotherapeutic vaccine was primarily designed to avoid undesired inflammatory side effects, both in mice and humans (without need of coadjuvant modification), while inducing effective antibody titer rates. We demonstrated that long-term immunization with EB101 induced A*β* antibodies in the preventive and the therapeutic treatment periods, which effectively reduced amyloid deposition in both cortical and hippocampal brain regions and prevented neuroinflammation, probably by blocking toxicity in the surrounding neurons. However, the AN1792 vaccine was shown to induce in 6% of the patients an inflammatory reaction that might have led to the encephalitis type reaction observed in the clinical trial, which was responsible for the immediate halt in those clinical studies. It seems that AN1792 immunization, delivered in Qs-21 and polysorbate 80 adjuvant, induced a Th1 type response that likely led to autoaggressive T cells and aseptic meningoencephalitis. In the present experiments, we demonstrated that a judiciously selected adjuvant produces a different humoral response [22]. Indeed, we have shown that our novel immunotherapeutic strategy boosts specific A*β*_1–42_ antibody generation and induces a Th2 noninflammatory immune response. This boosted effect is in accordance with previous immunotherapeutic studies [34]. Taking together all the present findings, these results suggest that EB101 vaccine can achieve, in a future clinical trial, immunogenic response rates quite similar to the AN1792 without the inflammatory effects reported. The immunohistochemical analysis obtained in the present study shows that all the EB101 immunized mice resulted in an almost complete prevention of A*β* deposition in the brain, with only a few compact A*β* plaques in the retrosplenial and dentate gyrus regions. Although there was a slightly increase in death in EB101 treated mice compared to controls, this significant reduction of A*β* plaques in EB101 treated mice was proven to be more efficient than that obtained with A*β*_42_ + CFA/IFA immunizations. A comparison of A*β* plaque reduction rates among treated groups surprisingly showed that other than EB101 and A*β*_42_ + CFA/IFA treated groups, some amyloid deposits were also reduced in Groups B (A*β*_1–42_/liposome) and C (S1P-containing liposomes). Although the reduction rates in these mice were markedly low, the subtle lowering effect might be due to the interaction between the immunization elements (A*β*_1–42_ and S1P) and the mouse immunogenic system, as reported by others [35]. Based on our present results, S1P, one of the components of the adjuvant used with EB101, might be responsible for the neuroprotective effect observed in the vulnerable neuronal areas of EB101 treated mice. The advantage in the A*β* load prevention/reduction by combining A*β*_1–42_ and S1P in a unique formulation vaccine has been also observed in the reduced hippocampal load of EB101 immunized mice (Gr A) when compared with different vaccine components (Gr B-C). In fact, S1P appears to have a demonstrated therapeutic potential as a regenerative agent in the nervous system since it controls migration of neuronal stem cells toward a site of spinal cord injury [36], induce cytoskeletal rearrangements to promote transmitter secretion [37], and also trigger diverse cellular effects including angiogenesis, cardiac development, immunity, cell motility, and neurite extension [38, 39]. Therefore, it is reasonable to expect that such a potent biologically active sphingolipid by itself may play an important role in the A*β* plaque reduction. In this study, the clearance efficiency rate of each treatment was analyzed and results suggested that EB101 (64/59%) appears to be more effective than A*β*_42_ + CFA/IFA (19/20%) vaccine in both preventive and therapeutic groups. The effectiveness of EB101 in older transgenic animals (18 months) showed a clearance effect of 59%, suggesting that EB101 may also have therapeutic potential in AD diagnosticated patient trials. Similar conclusion was reached by Wang and colleagues [40], who demonstrated that combination therapy in aged animals provides more valuable insights into a possible translation to humans. Our immunological measurements demonstrated once again that neuroinflammation develops in an A*β*-dependent fashion early in the course of AD [41] and can be alleviated or reversed by interventional A*β* vaccination such as EB101. Among the possible mechanisms of A*β* clearance proposed in AD immunotherapy [42], our results suggested that they may overlap and probably be disease state dependent, since the prevention and the therapeutic vaccine would likely benefit from the transport of A*β* antibodies into the CNS. However, some mechanisms of action such as the preventive effect of A*β* antibodies in the A*β* aggregation [43], microglial phagocytosis induced by the Fc binding portions between A*β* antibodies and microglia receptor [44], and the shift in the gradient of A*β* transport across the blood-brain barrier due to the increased concentration of A*β* antibodies in the vascular system resulting in an increase in efflux from the brain to blood [45, 46] may be playing a role in both preventive and therapeutic conditions. Our findings therefore suggest that novel treatment strategies aimed at maintaining or increasing microglial function and restoring the balance to the immunological system may represent an attractive therapeutic approach even at the advanced stages of AD. The immunization results obtained with EB101 during both preventive and therapeutic treatment showed no microhemorrhages, infiltration of lymphocytes or encephalitis-like reaction in the brain, suggesting that EB101 does not induce A*β*-specific Th1 reaction, possibly due to the neuroprotective effect of S1P. Delivering A*β*_1–42_ in a liposomal-S1P-adjuvant resulted in an effective immunological response including the induction of a Th2 T-cell response, which effectively prevented neuroinflammation in an animal model of AD as reported previously [24, 47]. Moreover, present results demonstrated that EB101 did not induce astrogliosis in APP/PS1 transgenic mice, whereas some GFAP-positive astrocyte clusters were aggregated in the affected brain areas of mice treated with A*β*_42_ + CFA/IFA. These results indicate that EB101 may result in an effective treatment in AD neuropathology due to the absence of peripheral proinflammatory cytokine secretion and the absence of astroglial activation in the brains of EB101 treated mice when compared with A*β*_42_ + CFA/IFA immunotherapeutic response. In a clinical setting, however, we may expect a wide differential response, in part depending upon the genomic profile of each patient [48, 49]. In AD behavioural studies, in particular among the motor coordination tests, the rotarod performance has been reported to be impaired in some AD transgenic mice models bearing A*β* plaques [50, 51]. This result was found in mutants prior to A*β* plaque formation, at 3 and 6 months of age, but was not reproduced after plaques had been formed, at 16 and 24 months of age [52], perhaps because of the declining abilities of older normal mice. Here we present the effects of vaccine treatments in APP/PS1 mice on motor coordination, a neglected aspect of the AD experimental behavior but justified on the basis of the deficient postural control reported in patients with Alzheimer's disease [53], sometimes even during early stages [54]. Our longitudinal motor behavioral results showed no significant differences in the ambulatory movement, grip strength, or coordination, addressed by their performance in the rotating rod between APP/PS1 and wild-type mice for any age group studied, in contrast with other treated groups, demonstrating that longitudinal motor assessments of AD transgenic mouse models can provide valuable information with regard to the effect of immunization on the progression of behavioral deficits and overall motor activity. As shown, the EB101-treated group of mice exhibited longer latencies and better balancing skills, indicating the absence of any negative effect of EB101 immunization on these mice. The classical fear conditioning test is assumed to reveal the functional integrity of the hippocampus [55], representing a special memory task to evaluate the AD models and its improvement after immunization periods. In this APP/PS1 mouse model, the first associative learning and spatial working memory deficits are shown at 5-6 months of age [56], although its impairment progression is relatively slow compared to most other models. At the end of preventive immunization period, EB101 and A*β*_42_ + CFA/IFA immunized mice have significantly shorter escape latencies than the other treatment groups (A*β*_42_/liposome, S1P-containing liposomes) and the controls in the fear conditioning test. Shorter escape latencies during spatial learning indicated that deficits of spatial memory were attenuated in EB101 and A*β*_42_ + CFA/IFA immunized mice. Previous studies have showed that by 15 months of age the spatial working memory is consistently impaired throughout the rest of the life span [31, 57]. At the end of therapeutic immunization period, both EB101 and A*β*_42_ + CFA/IFA immunized mice showed significantly improved results on the hippocampus-dependent spatial learning and memory test when compared with other treatment groups and the controls. Taken together, these observations are in agreement with the impairment in conditioned learning in response to a tone stimulus of aged APP/PS1 mice, although when immunized with EB101 during preventive or therapeutic periods, such impairments are significantly attenuated.

In summary, the present results corroborate our previous published findings showing that EB101 immunization reduces amyloid accumulation in the APP/PS1 mouse brain [24, 25]. The strongest reduction in the preventive immunization was observed in the retrosplenial cortex, followed by the hippocampus, and entorhinal-piriform cortex. Our results further support that EB101 is more effective when the mice are immunized prior to developing plaques in the hippocampal and cortical brain regions. There is extensive evidence in mouse preclinical studies [58] and human clinical trials [59, 60] that the adverse effects of active and passive A*β* vaccines result in microhemorrhages and encephalitic reactions [60–63], yet the mechanism by which they occur has not been fully elucidated. In the treatment sections analyzed, we found no microhemorrhages or acute inflammatory processes in all the survived mice of the group, indicating that EB101 therapeutic approach presents no adverse inflammatory effects in this AD mice models. Thus, our present results unequivocally demonstrate that EB101 vaccine may not only halt AD-related pathology as a preventive treatment but may also reverse it in established cases, related to the improvement of the learning and memory, in both cases leading to a robust immunological therapeutic response. Present experiments also demonstrate that EB101 exerts more significant and substantial effects on the reduction of A*β* deposition than A*β*_42_ + CFA/IFA vaccine in A*β* mice models of AD. However, further exploration of the contribution of genetic, environmental, and physiological factors in the development resistance to endogenous A*β*-antibody is needed in order to optimize and modulate the therapeutic strategies currently being developed.

## 5. Conclusions

The present study, carried out in APP/PS1 transgenic mouse model of AD, compared the A*β* burden reduction and cellular immune responses produced by two immunization delivery strategies of A*β*: in Freund adjuvant (described by Webster et al., [10]) and in liposome/S1P matrix (described by Schenk et al., [17]). The EB101 immunotherapeutic strategy boosted antibody generation, A*β* plaque reduction, and an anti-inflammatory response more effectively than A*β*_42_ + CFA/IFA, especially if given prior to or in the early stages of pathology. The present results also showed that vaccination with EB101 prevented the formation of new plaques and, presumably, led to a reduction in established plaques while inducing a Th2 immune reaction without encephalitogenic T-cell responses or brain inflammation related with astrogliosis. However, despite the efficacy demonstrated of this novel vaccine approach, further preclinical and clinical studies on EB101 are warranted to evaluate its efficacy and the mechanism for the future utility of such vaccine for AD therapy.

## Figures and Tables

**Table 1:** Summary of antibodies used.

Antibody	Dilution	Immunogen	Source	Labeling	Clone name	Type	Catalog number	Lot number	Ref.
B-Amyloid	1; 1000	KLH-conjugated synthetic peptide corresponding aa 36–42 of human amyloid Bx-42 peptide	Millipore	Amyloid-Bx-42 polypeptide	12F4	Mouse monoclonal	Number 05-831	DAM1447121	[24, 25, 27]
GFAP	1; 400	Antiglial fibrillary acid protein (mouse IgG1 isotype)	Sigma Aldrich	Antiglial fibrillary acid protein	G-A-5	Mouse monoclonal	Number G3893	078K4830	[9, 24, 25, 28, 29]
DNs	1; 300	Degenerating plaque neurites	Millipore	Degenerating plaque neurites and neurofibrillary tangles	—	Rabbit polyclonal	Number AB1518	PS01434496	[24, 25, 27]

## List of Abbreviations

AD: Alzheimer's disease
A*β*: *β*-Amyloid protein
APP: Amyloid precursor protein
APT: Anterior pretectal nucleus
CA1: Field CA1 hippocampus
CA3: Field CA3 hippocampus
CPu: Caudate putamen (striatum)
Cx: Cortex
DG: Dentate gyrus
DN: Dystrophic neurite
Ect: Entorhinal cortex
CFA: Complete Freund's adjuvant
GrDG: Granular dentate gyrus
IFA: Incomplete Freund's adjuvant
IL: Interleukin
LPMR: Lateral posterior thalamic nucleus (mediorostral)
MoDG: Molecular dentate gyrus
Pir: Piriform cortex
PoDG: Polymorph dentate gyrus
RSGb: Retrosplenial granular cortex b region
Tg: Transgenic
Th: T helper.
